# NSUN2 Negatively Regulates TP53 mRNA Stability to Promote the Malignant Progression of Nasopharyngeal Carcinoma

**DOI:** 10.3390/cancers17243950

**Published:** 2025-12-10

**Authors:** Lemei Zheng, Jianxia Wei, Xiaolong Li, Mengna Li, Changning Xue, Shipeng Chen, Qingqing Wei, Songqing Fan, Wei Xiong, Ming Zhou, Hongyu Deng

**Affiliations:** 1 NHC Key Laboratory of Carcinogenesis, Hunan Key Laboratory of Oncotarget Gene, Hunan Cancer Hospital and the Affiliated Cancer Hospital of Xiangya School of Medicine, Central South University, 283 Tongzipo Road, Changsha 410013, China; 246501048@csu.edu.cn (L.Z.); 226501026@csu.edu.cn (J.W.); limengna@hnca.org.cn (M.L.); 236501044@csu.edu.cn (C.X.); chensp@csu.edu.cn (S.C.); 256501051@csu.edu.cn (Q.W.); xiongwei@csu.edu.cn (W.X.); 2 Cancer Research Institute, School of Basic Medical Sciences, Central South University, 110 Xiangya Road, Changsha 410078, China; 248211170@csu.edu.cn; 3 The Key Laboratory of Carcinogenesis and Cancer Invasion of the Chinese Ministry of Education, Central South University, Changsha 410078, China; 4 Department of Pathology, The Second Xiangya Hospital, Central South University, Changsha 410011, China; songqingfan@csu.edu.cn

**Keywords:** NSUN2, m5C, TP53, nasopharyngeal carcinoma, malignant progression

## Abstract

**Background:** Nasopharyngeal carcinoma (NPC) is a prevalent malignancy in China, often diagnosed at advanced stages. The 5-methylcytosine (m5C) RNA modification plays a crucial role in tumorigenesis, influencing cell proliferation, differentiation, metabolism, invasion, metastasis, and immune evasion. NSUN2, a key m5C methyltransferase, has been implicated in various cancers, but its role in NPC remains unclear. **Methods:** NSUN2 expression in NPC tissues was explored by bioinformatics analysis, qPCR, Western blot, and immunohistochemistry. Functional roles of NSUN2 in proliferation, apoptosis, migration, and invasion were evaluated in NPC cell lines using CCK-8, colony formation, flow cytometry, wound-healing and transwell assays. The mechanism by which NSUN2 regulates TP53 was investigated by m5C-RIP, RNA stability assays, dual-luciferase reporter assay and rescue experiments. The NSUN2/TP53 axis was further validated in vivo through xenograft mouse models. **Results:** NSUN2 is significantly overexpressed in NPC tissues, and its high expression correlates with poor prognosis. Functional assays reveal that NSUN2 promotes NPC cell proliferation, migration, and invasion and inhibits apoptosis. Mechanistically, NSUN2 negatively regulates TP53 expression by increasing m5C modification at the CDS 1228 site, thereby decreasing TP53 mRNA stability and expression. Knockdown of TP53 counteracts the inhibitory effects of NSUN2 knockdown on proliferation, migration, and invasion in NPC cells. Additionally, in vivo experiments revealed that NSUN2 knockdown suppresses tumor growth in xenograft models, while TP53 knockdown reverses the growth-inhibitory effect of NSUN2 knockdown on xenograft tumors. **Conclusions:** Our findings indicate that NSUN2 partially negatively regulates TP53 mRNA stability, promoting malignant progression and acting as an oncogene in NPC by downregulating TP53 through m5C modification. Thus, targeting the NSUN2/TP53 axis could be a potential therapeutic strategy for NPC.

## Simple Summary

Nasopharyngeal carcinoma (NPC) is a prevalent malignancy in China, often diagnosed at advanced stages. In this study, we identified high NSUN2 expression in NPC tissues, Mechanistically, NSUN2 negatively regulates TP53 expression by increasing m5C modification at the CDS 1228 site, thereby decreasing TP53 mRNA stability and expression. Knockdown of TP53 counteracts the inhibitory effects of NSUN2 knockdown on proliferation, migration, and invasion in NPC cells. Additionally, in vivo experiments revealed that NSUN2 knockdown suppresses tumor growth in xenograft models, while TP53 knockdown reverses the growth-inhibitory effect of NSUN2 knockdown on xenograft tumors. Our findings indicate that NSUN2 partially mediated through the negative regulation of TP53 mRNA stability, promoting malignant progression and acting as an oncogene in NPC by downregulating TP53 through m5C modification. Thus, targeting the NSUN2/TP53 axis could be a potential therapeutic strategy for NPC.

## 1. Introduction

Nasopharyngeal carcinoma (NPC) is the most common head and neck malignancy in southern China [1,2]. Due to its concealed primary site, NPC is often difficult to detect in its early stages [3,4,5,6]. Therefore, investigating the molecular mechanisms underlying NPC and identifying novel therapeutic targets are crucial for improving patient prognosis. RNA modifications, particularly 5-methylcytosine (m5C), have been closely linked to tumorigenesis [7]. The m5C modification is dynamically regulated by methyltransferases (Writers, exemplified by NSUN family proteins), reversibly removed by demethylases (Erasers, such as TET2), and functionally interpreted by recognition proteins (Readers, including ALYREF). This modification confers RNA structural stability to resist degradation, enhances mRNA translation initiation efficiency, governs RNA subcellular localization through mechanisms such as nuclear export mediation, and modulates critical processes including embryonic development, cell differentiation, and metabolic homeostasis [8,9,10]. Increasing evidence indicates that m5C modification plays a key role in cancer development [11], particularly by promoting radiotherapy resistance through enhanced DNA repair processes [12,13]. Furthermore, m5C modification has been implicated in the prognosis of lung squamous cell carcinoma and head and neck squamous cell carcinoma (HNSC), suggesting that m5C regulatory molecules may be pivotal in cancer progression [14,15]. Therefore, exploring the role of m5C modification in NPC may provide novel insights and therapeutic strategies.

NOP2/Sun RNA methyltransferase 2 (NSUN2) is a key methyltransferase responsible for m5C modification and is capable of methylating both tRNA and mRNA. NSUN2 catalyzes the methylation of cytosine at the fifth carbon position within mRNA [16]. Studies have shown that NSUN2 plays a significant role in tumorigenesis [17,18]. In gastric cancer (GC), NSUN2 promotes cell proliferation and metastasis, with similar pro-tumorigenic effects observed in other malignancies, including hypopharyngeal squamous cell carcinoma and prostate cancer [17,19,20,21,22,23]. In gliomas, NSUN2 could methylate autotaxin mRNA, thus increasing autotaxin protein expression and promoting tumor cell migration [24]. Additionally, NSUN2 is involved in immune regulation by methylating and upregulating IL-17A mRNA, inhibiting the cGAS/STING pathway, and driving tumorigenesis [25]. NSUN2 also participates in ferroptosis regulation [26]. It is associated with poor prognosis in liver cancer, clear cell renal carcinoma, and lung adenocarcinoma and is considered an adverse prognostic factor in GC and HNSC [27,28]. Our previous research found that NSUN2 is highly expressed in various tumors and serves as a potential prognostic marker. In NPC, NSUN2 promotes cell proliferation [29]. However, the specific functions and mechanisms by which NSUN2 contributes to NPC remain unclear. Therefore, investigating m5C-related molecules, particularly NSUN2, is essential for understanding NPC development and identifying therapeutic approaches.

TP53 plays a critical role in inhibiting tumorigenesis. As a transcription factor, TP53 suppresses tumor formation through various mechanisms, including cell cycle arrest, autophagy, metabolic reprogramming, apoptosis, senescence, DNA repair, and immune surveillance [30,31,32,33,34,35]. For instance, TP53 plays a role in immune regulation by modulating Toll-like receptor 8 [36]. Notably, in prostate cancer, gene set enrichment analysis has revealed that NSUN2 is enriched in the TP53 signaling pathway [37]. In this study, we found that NSUN2 negatively regulates TP53 expression in NPC. Given the challenges associated with targeting TP53 directly, understanding its upstream regulatory mechanisms may provide new therapeutic strategies.

In this study, we demonstrated that NSUN2 is highly expressed in NPC and correlates with poor prognosis. NSUN2 overexpression promotes NPC cell proliferation and clonogenic ability, inhibits apoptosis, and enhances migration and invasion. Mechanistic studies revealed that NSUN2 negatively regulates TP53 mRNA stability and expression by suppressing the m5C modification at the coding sequence (CDS) 1228 site. Additionally, TP53 knockdown reverses the inhibitory effects caused by NSUN2 knockdown in NPC cells. Therefore, the NSUN2/TP53 axis represents a potential therapeutic target for NPC.

## 2. Materials and Methods

### 2.1. Clinical Samples

All normal nasopharyngeal tissues and NPC tissues used in this study were provided by the Pathology Department of the Second Xiangya Hospital. Among the samples, five normal tissues and five NPC biopsy samples were fresh tissues from patients. Total RNA and protein were isolated from the same five tissue samples and were used for the detection of NSUN2 mRNA and protein expression. Additionally, 36 normal and 76 NPC tissues were paraffin-embedded and used for the immunohistochemical assessment of NSUN2 protein expression. All samples were collected with informed patient consent, evaluated by pathologists, and approved by the institutional review board of the School of Basic Medical Science at Central South University (2022-KT148), following local guidelines.

### 2.2. Cell Culture

The CNE2 and 5-8F cell lines, sourced from the Cell Center of Central South University (Changsha, China), were maintained in Dulbecco’s modified Eagle’s medium (Life Technologies, Carlsbad, CA, USA) containing 10% fetal bovine serum (BI, Jerusalem, Israel), penicillin (100 U/mL), and streptomycin (100 μg/mL) at 37 °C. All cell lines were routinely verified to be free of mycoplasma contamination.

### 2.3. Plasmids and Construction of Stable Cell Lines

Using the pLVX-shRNA1 vector, pLVX-shNSUN2 and pLVX-shTP53 knockdown plasmids were constructed through double enzyme digestion and ligation with their respective silence sequences. Additionally, the NSUN2 overexpression recombinant vector was created by fusing the NSUN2 CDS region to pcDNA3.1 (pcDNA3.1/NSUN2). The pcDNA3.1-3×Flag-NSUN2-MUT plasmid was purchased from Youbio Biological Technology Co., Ltd. (Changsha, China). All these plasmids were utilized to generate stable cell lines in CNE2 and 5-8F through lentiviral transduction. The sequences for NSUN2 and TP53 silencing are listed in Table 1.

### 2.4. Western Blot Analysis

CNE2 and 5-8F cells were harvested 48 h post-transfection for protein extraction. Proteins were lysed with radioimmunoprecipitation assay lysis buffer (New Cell & Molecular Biotech, Suzhou, China), along with the addition of protease inhibitors and PMSF, and protein concentrations were measured prior to electrophoresis and transfer. The membrane was blocked with 5% non-fat milk in TBST for 1 h at room temperature with gentle agitation. Subsequently, primary antibodies against NSUN2 (1:2000, Proteintech, Wuhan, China), TP53 (1:2000, Proteintech), GAPDH (1:5000, Proteintech), phosphatase and tensin homolog (PTEN) (1:500, Bioworld, Beijing, China), tight junction protein 1 (ZO-1) (1:1000, ABclonal, Wuhan, China), vimentin (1:1000, ABclonal), Bcl2-associated X protein (BAX) (1:1000, Cell Signaling Technology, Danvers, MA, USA), BCL2 apoptosis regulator (Bcl2) (1:500, Proteintech), cyclin dependent kinase 4 (CDK4) (1:1000, ABclonal), and cyclin dependent kinase inhibitor 1A (p21) (1:500, CST) were applied overnight. Following three 15 min washes with TBST buffer, the membrane was incubated with an HRP-conjugated secondary antibody (1:5000, Proteintech) at 37 °C for 1 h, and proteins were visualized using enhanced chemiluminescence.

### 2.5. Cell Counting Kit-8 (CCK-8) Assay

Twenty-four hours post-transfection, CNE2 and 5-8F cells were trypsinized, centrifuged, and counted. A total of 100 μL containing 1000 cells was seeded into each well of a 96-well plate, with four replicates per group, and cultured. After cell attachment, 10 μL of CCK-8 reagent (Selleck Chemicals, Houston, TX, USA) was added in the dark and incubated at 37 °C for 2 h. Absorbance was measured at 450 nm using a microplate reader. Data were analyzed and visualized using GraphPad Prism 8.0 (GraphPad Software, La Jolla, CA, USA).

### 2.6. Colony Formation Assays

Twenty-four hours after transfection, CNE2 and 5-8F cells were trypsinized, centrifuged, and counted. Cell suspensions containing 2000 cells were plated in culture dishes with three replicates per group and incubated until visible colonies formed. The colonies were fixed with 4% paraformaldehyde for 15 min and stained with crystal violet (Beyotime, Beijing, China) for 15–20 min. The plates were then washed with distilled water, air-dried, scanned, and stored.

### 2.7. Migration and Invasion Assays

For the migration assay, transfected CNE2 and 5-8F cells were scratched using a pipette tip, and 1% or 2% serum medium was added to observe the migration of NPC cells at 0, 24, and 48 h. Cell invasion was assessed using Matrigel-coated Transwell chambers. Matrigel (BD, Franklin Lakes, NJ, USA) was added to chambers for 2 h, and transfected CNE2 and 5-8F cells were seeded in chambers (Corning Inc., Corning, NY, USA), with 20% serum medium placed in the lower chamber. After 48 h, cells were stained with crystal violet and cleaned. The number of cells that migrated through the Matrigel matrix and the membrane pores to the lower chamber was quantified by counting cells in multiple random microscopic fields.

### 2.8. Flow Cytometry-Mediated Apoptosis Assay

Transfected CNE2 and 5-8F cells were cultured in serum-free medium for 24 h, then harvested and washed with PBS one to two times. Cells were resuspended in 1× binding buffer (diluted 10× binding buffer with deionized water according to 1:9) and stained with 5 μL Annexin V and 5 μL propidium iodide in the dark at room temperature (Chamot Biotechnology, Shanghai, China). The samples were filtered into flow cytometry tubes and analyzed using flow cytometry (Cytek DxP Athena). Data were analyzed using the FlowJo 10.8.1 software (FlowJo LLC, Ashland, OR, USA), with experiments performed in triplicate.

### 2.9. RNA Extraction

Transfected CNE2 and 5-8F cells grown in six-well plates were lysed on ice using Trizol (Invitrogen, Waltham, MA, USA) for 3–5 min. After adding chloroform, the samples were centrifuged, and the upper aqueous phase was transferred to a new tube. Isopropanol was added, and RNA was precipitated at −20 °C for 30 min. After centrifugation, RNA was washed with 75% ethanol, air-dried, and dissolved in RNase-free water. RNA concentrations were measured, and samples were stored at −80 °C. Reverse transcription and reverse transcription quantitative PCR (RT-qPCR) were then performed.

### 2.10. Actinomycin D Experiment

Twenty-four hours after transfection of CNE2 and 5-8F cells, 2 μL of actinomycin D (1 mg/mL) (Selleck Chemicals) was added at various time points (0, 2, 4, 8, 16 h), and CNE2 and 5-8F cells were incubated at 37 °C. RNA was extracted and reverse transcribed using the reverse transcription kit (Vazyme Biotech Co., Ltd., Nanjing, China), and mRNA stability was analyzed using RT-qPCR with 18S ribosomal RNA as an internal control.

### 2.11. RNA Binding Protein Immunoprecipitation (RIP) Assay

Antibodies (NSUN2 (Proteintech), 2 μL; m5C (Abcam, London, UK), 4 μL; IgG (Proteintech)) were bound to Protein A and G beads (Selleck Chemicals) for 2–3 h. Cell pellets were cross-linked with 37% formaldehyde and quenched with 2 M glycine. Cells were lysed with IP lysis buffer (containing 20 μL 0.1 M PMSF, 20 μL protease inhibitors (100×) diluted to 1×, and 10 μL RNase inhibitor (Accurate Biology, Changsha, China; Recombinant RNase Inhibitor) (40 U/μL)). Lysates were incubated with beads at 4 °C overnight (16–20 h), washed, and incubated with RIP buffer (100 mM NaCl, 50 mM Hepes, 5 mM EDTA, 10 mM DTT, 0.5% Triton X-100, 10% glycerol, 1% SDS) and RNase inhibitors. Samples and input controls were incubated at 70 °C for 1 h to reverse cross-links. Beads were centrifuged, supernatants collected, mixed with Trizol, and RNA extracted, reverse transcribed, and analyzed via qPCR.

### 2.12. Dot Blot Assay

RNA samples (400 ng/μL) from CNE2 and 5-8F cells transfected with pcDNA3.1/NSUN2 or siNSUN2 were denatured at 95 °C for 3 min to disrupt secondary structures. Two microliters of RNA were spotted onto a nylon membrane, air-dried, and UV-cross-linked at 254 nm for 1 h. The membrane was blocked with 5% skim milk, incubated with primary antibody (NSUN2, Proteintech) overnight, and detected using ECL. The membrane was stained with methylene blue and washed before imaging.

### 2.13. Dual-Luciferase Reporter Assay

PGL3-Promoter reporter plasmids (PGL3-Promoter/TP53 mRNA CDS + 3′UTR, PGL3-Promoter/TP53 mRNA CDS, PGL3-Promoter/TP53 mRNA 3′UTR, PGL3-Promoter/TP53 mRNA CDS 1051 Mut, PGL3-Promoter/TP53 mRNA CDS 1228 Mut, PGL3-Promoter/TP53 mRNA CDS 1051 + 1228 Mut), target plasmids for NSUN2 or pLVX-shNSUN2, and internal control Renilla plasmids were co-transfected into cells. Forty-eight hours later, cells were lysed, and luciferase activity was measured using a dual-luciferase reporter assay system (Vazyme). The firefly luciferase activity was normalized to Renilla luciferase, and the ratio was used to analyze differences between groups.

### 2.14. Immunohistochemistry (IHC)

Tissues were deparaffinized, rehydrated, and subjected to antigen retrieval. The antigen retrieval was performed by microwave treatment in EDTA buffer (pH 9.0). Endogenous peroxidase activity was blocked, and tissues were incubated with anti-NSUN2 (1:1000, Proteintech), anti-TP53 (1:500, Proteintech), anti-cleaved-PARP (c- PARP) (1:100, Proteintech), and anti-MKI67 (Ki67) (1:100, Bioworld). Secondary antibodies were applied, followed by DAB staining and hematoxylin counterstaining. Tissues were imaged and analyzed according to the product of distribution score of positive rate and intensity score [38,39].

### 2.15. Nude Mouse Xenograft Model

Animal experiments were approved by the Ethics Committee of Central South University (CSU-2023-0519). Three- to four-week-old nude mice were purchased from Hunan Slake Jingda Experimental Animal Co., Ltd. (Changsha, China) and acclimated in an SPF laboratory for 1 week. We divided 15 BALB/c nude mice into three groups: CNE2/Ctrl, CNE2/shNSUN2, and CNE2/shNSUN2 plus shTP53, with each group containing five mice. A total of 5 × 10^6^ cells in a 0.9% saline solution with Matrigel (total volume of 150 μL) were subcutaneously injected into the axilla of each mouse, and tumor growth was monitored every 2 days. Tumor volume was calculated using the following equation: volume = (length × width^2^)/2. After euthanasia, tumors were excised, weighed, fixed in 4% paraformaldehyde, and finally embedded in paraffin for IHC staining and expression analysis. The size of the tumor in the experiment met the ethical standards.

### 2.16. Gene Expression and Clinical Correlation Analysis

TCGA-HNSC RNA-seq dataset and GSE12452 dataset (treatment-naïve) were acquired from the publicly available TCGA and GEO databases, respectively. Differential expression analysis was performed using R (v4.0.5). Raw expression matrices were processed through the following pipeline: (1) normalization and conversion to transcripts per million values, (2) differential expression analysis using DESeq2 (for TCGA-HNSC) and limma (for GSE12452) packages, and (3) visualization of results via pheatmap (heatmaps) and ggplot2 (boxplots) packages. Gene and protein expression analysis, as well as the correlation between gene expression and clinical progression and staging, were obtained from the UALCAN database (https://ualcan.path.uab.edu/).

### 2.17. Statistical Analysis

All experiments were carried out independently. Data were analyzed using GraphPad Prism 8.0. Student’s *t*-test was used to compare two groups, while one-way ANOVA was employed for multiple group comparisons. Colony formation, scratch, and transwell data were analyzed using ImageJ software (version 1.53). Data were presented as mean ± standard deviation or mean ± standard error of the mean. Chi-square tests were performed to assess the relationship between NSUN2 expression and clinical characteristics. Statistical significance was set at * for *p* < 0.05, ** for *p* < 0.01, *** for *p* < 0.001, and ns for non-significant *p* > 0.05.

## 3. Results

### 3.1. High NSUN2 Expression in NPC and Its Association with Poor Prognosis

To investigate the role of m5C modification-related molecules in NPC, we analyzed the expression of m5C-associated “writers,” “readers,” and “erasers” between normal and tumor samples using the GEO dataset GSE12452 and TCGA-HNSC dataset through R language. In the GSE12452 dataset, ALYREF, NSUN1, NSUN2, and YBX1 were significantly upregulated in NPC, whereas NSUN7 was downregulated. Similarly, in TCGA-HNSC dataset, NSUN5, NSUN2, and NSUN1 were overexpressed in NPC, while NSUN7 and TET2 were downregulated (Figure S1A,B). To identify the most significantly differentially expressed m5C-related molecules in NPC, we found that NSUN1 and NSUN2 were consistently upregulated, and NSUN7 was downregulated in both datasets (Figure S1C). Given that NSUN2 is a key methyltransferase involved in m5C modification and regulates m5C modification in mRNA, we further investigated its protein expression in NPC and discovered significantly elevated NSUN2 levels (Figure S1D), which correlated with clinical progression and staging (Figure S1E,F). These findings suggest that NSUN2 may play a critical role in NPC development.

**Figure 1:**
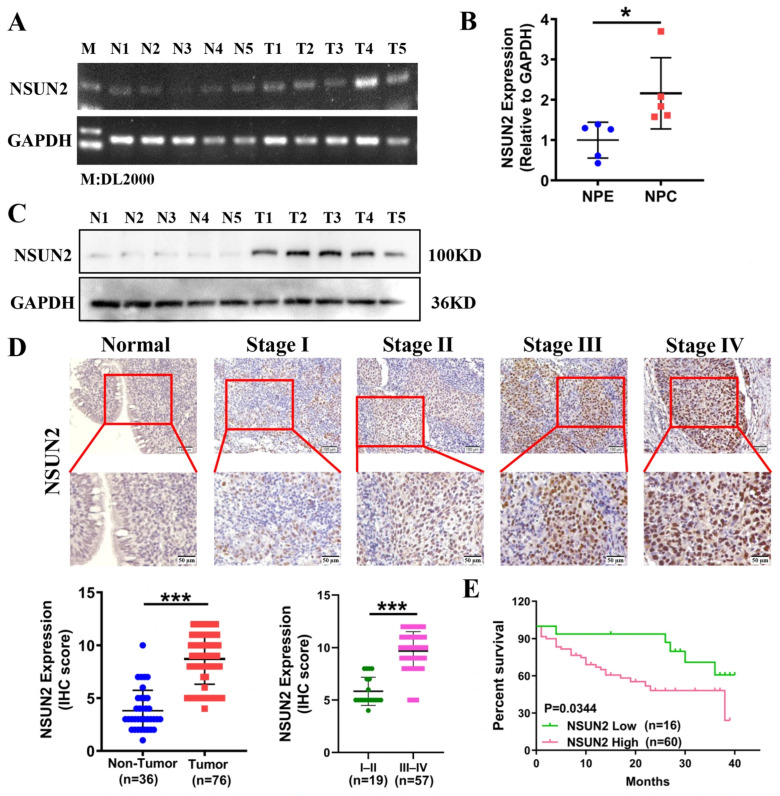
NSUN2 is highly expressed in nasopharyngeal carcinoma tissues and correlates with poor prognosis. (**A**) Gel electrophoresis analysis of NSUN2 mRNA expression. (**B**) qPCR analysis of NSUN2 mRNA expression in normal and tumor tissues. (**C**) Western blot analysis of NSUN2 protein expression in tissues. (**D**) Immunohistochemistry detection of NSUN2 expression in patient samples (Scale bar: up: 100 µm; Down: 50 µm). (**E**) Prognostic analysis of NSUN2 expression in tissue samples (based on the median split of NSUN2 expression). NPE: normal nasopharyngeal epithelial tissue; NPC: nasopharyngeal carcinoma tissue. *, *p* < 0.05; ***, *p* < 0.001. The uncropped bolts are shown in Supplementary Materials.

Furthermore, we assessed NSUN2 mRNA and protein expression in the same 5 NPE and 5 NPC tissue samples. Following RNA extraction, PCR amplification, and qPCR analysis, NSUN2 mRNA levels were significantly elevated in NPC compared with normal tissues (Figure 1A,B). Moreover, we examined the protein expression of NSUN2 using Western blot and IHC. Western blot analysis of five normal and five tumor tissues revealed elevated NSUN2 protein levels in NPC tissues. IHC analysis of 36 normal and 76 NPC tissues further confirmed higher NSUN2 protein expression, which correlated with clinical stage (Figure 1C,D). Kaplan–Meier survival analysis demonstrated that elevated NSUN2 expression was associated with poor prognosis (Figure 1E). Additionally, NSUN2 expression was significantly correlated with clinical stage, tumor size, and lymph node metastasis but showed no significant association with gender or age (Table 2).

### 3.2. NSUN2 Promotes Proliferation, Migration, and Invasion, and Reduces NPC Cell Apoptosis

**Figure 2:**
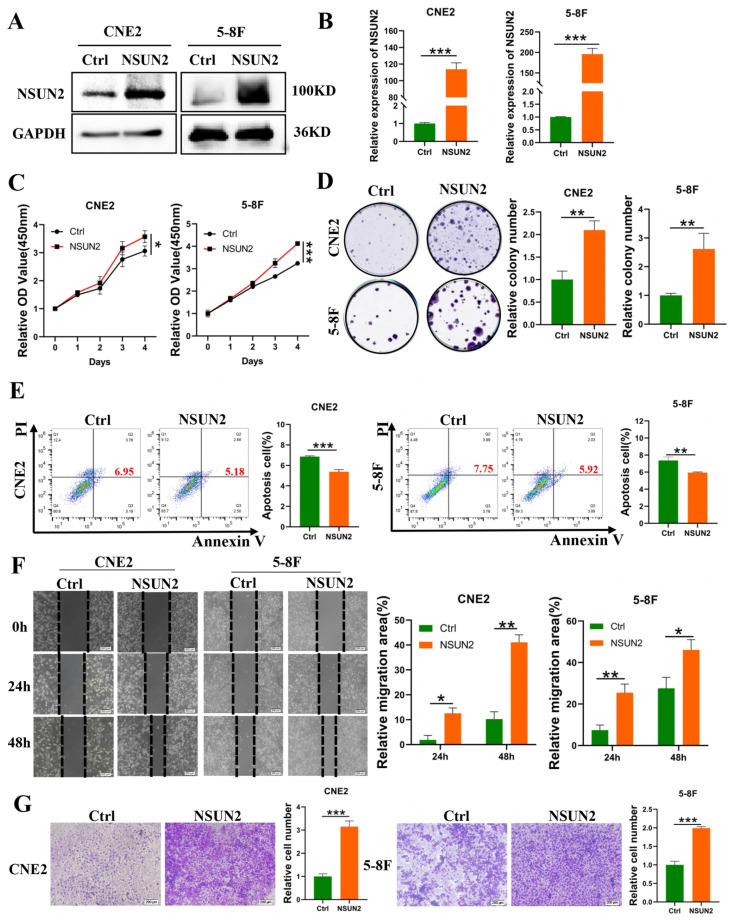
NSUN2 promotes proliferation, migration and invasion, and reduces apoptosis of NPC cells. (**A**) Western blot analysis of NSUN2 overexpression efficiency. (**B**) qPCR analysis of NSUN2 overexpression efficiency. (**C**) CCK-8 assay evaluating the effect of NSUN2 overexpression on the proliferation of CNE2 and 5-8F cells. (**D**) Clonogenic assay evaluating the effect of NSUN2 overexpression on colony formation in CNE2 and 5-8F cells. (**E**) Flow cytometry analysis of the effect of NSUN2 overexpression on apoptosis in NPC cells (The red numbers represent the total apoptosis rate). (**F**) Scratch assay evaluating the effect of NSUN2 overexpression on cell migration. (**G**) Invasion assay assessing the effect of NSUN2 overexpression on cell invasion. Data are representative of three independent experiments. *, *p* < 0.05; **, *p* < 0.01; ***, *p* < 0.001. The uncropped bolts are shown in Supplementary Materials.

To investigate the effects of NSUN2 on NPC cell biology, we overexpressed NSUN2 in CNE2 and 5-8F cells. Western blot and qPCR assays confirmed successful overexpression (Figure 2A,B). CCK-8 and colony formation assays revealed that NSUN2 overexpression significantly enhanced cell proliferation and colony formation (Figure 2C,D). Starvation-induced apoptosis, as measured by flow cytometry, showed that NSUN2 overexpression reduced apoptosis rates (Figure 2E). Wound healing and invasion assays demonstrated that NSUN2 overexpression significantly promoted cell migration and invasion (Figure 2F,G). These findings indicate that NSUN2 exerts oncogenic roles in NPC progression.

**Figure 3:**
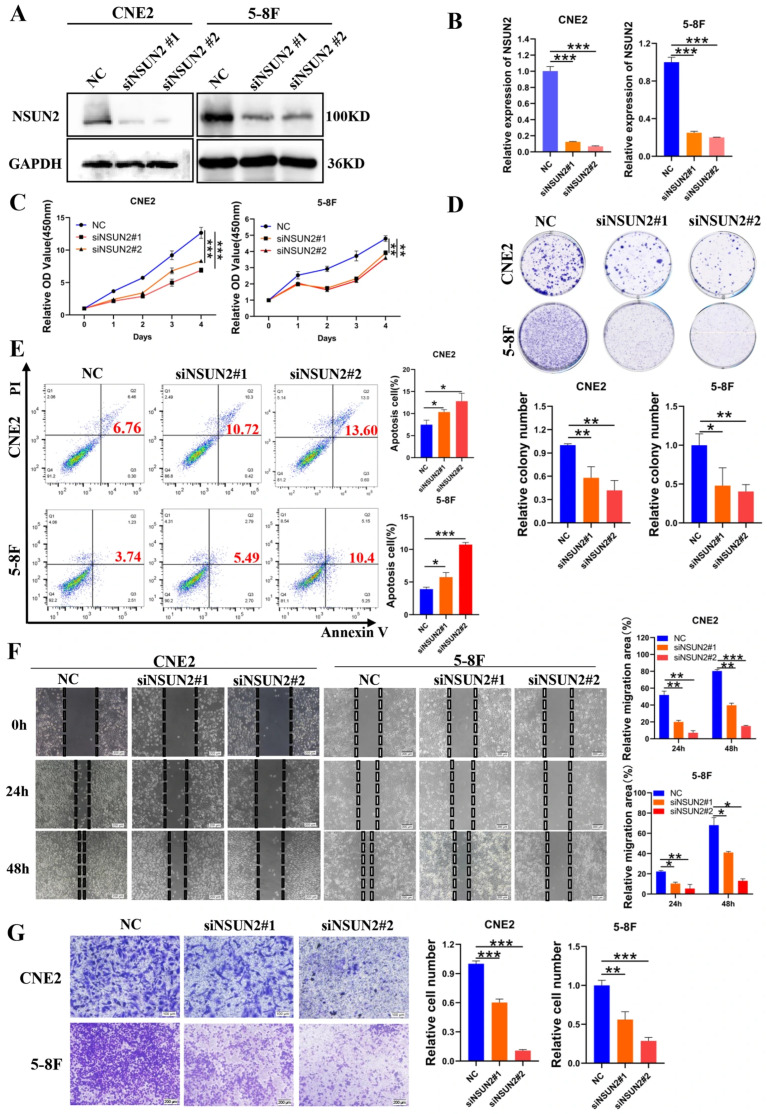
Silencing NSUN2 inhibits proliferation, migration, and invasion and promotes apoptosis in nasopharyngeal carcinoma cells. (**A**) Western blot analysis of NSUN2 silencing efficiency. (**B**) qPCR analysis of NSUN2 silencing efficiency. (**C**) CCK-8 assay evaluating the effect of NSUN2 silencing on the proliferation of CNE2 and 5-8F cells. (**D**) Clonogenic assay evaluating the effect of NSUN2 silencing on colony formation in CNE2 and 5-8F cells. (**E**) Flow cytometry analysis of NSUN2 silencing on apoptosis in NPC cells (The red numbers represent the total apoptosis rate). (**F**) Scratch assay evaluating the effect of NSUN2 silencing on cell migration. (**G**) Invasion assay assessing the effect of NSUN2 silencing on cell invasion. Data are representative of three independent experiments. *, *p* < 0.05; **, *p* < 0.01; ***, *p* < 0.001. The uncropped bolts are shown in Supplementary Materials.

To further investigate the effect of NSUN2 in the progression of NPC, we evaluated the silencing efficiency of NSUN2 using Western blot and qPCR (Figure 3A,B). CCK-8 and colony formation assays demonstrated that NSUN2 knockdown significantly inhibited cell proliferation and colony formation (Figure 3C,D). Starvation-induced apoptosis, as measured by flow cytometry, showed that NSUN2 silencing also significantly increased apoptosis rates (Figure 3E). Moreover, wound healing and invasion assays revealed that NSUN2 silencing suppressed cell migration and invasion (Figure 3F,G), indicating that NSUN2 silencing can inhibit the malignant phenotype of NPC cells.

### 3.3. NSUN2 Negatively Regulates TP53 Expression via m5C Modification

**Figure 4:**
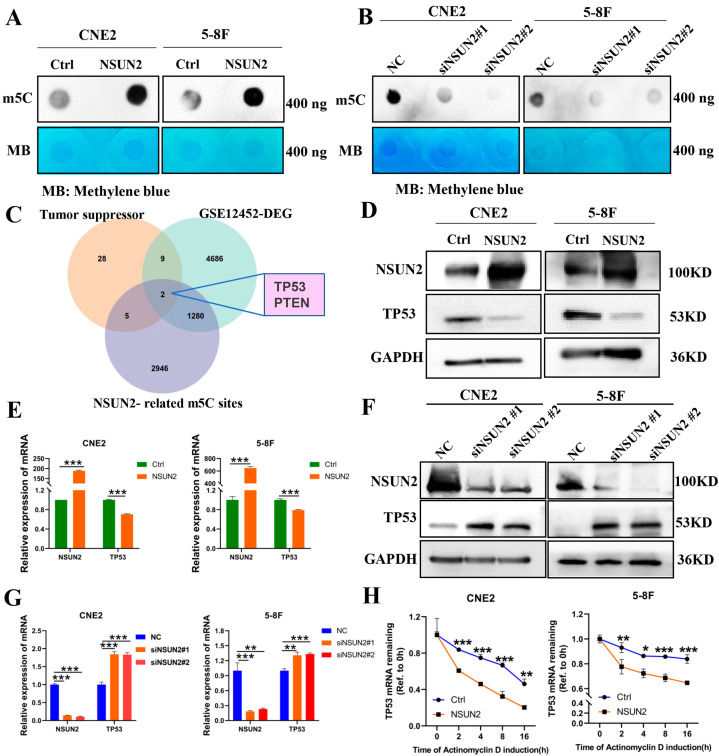
NSUN2 negatively regulates TP53 expression. (**A**) Effect of NSUN2 overexpression on m5C modification in CNE2 and 5-8F cells. (**B**) Effect of NSUN2 silencing on m5C modification in CNE2 and 5-8F cells. (**C**) Screening of potential downstream genes of NSUN2 in NPC. (**D**) Effect of NSUN2 overexpression on TP53 protein levels. (**E**) Effect of NSUN2 overexpression on TP53 mRNA levels. (**F**) Effect of NSUN2 silencing on TP53 protein levels. (**G**) Effect of NSUN2 silencing on TP53 mRNA levels. (**H**) Effect of NSUN2 on TP53 mRNA stability in CNE2 and 5-8F cells. *, *p* < 0.05; **, *p* < 0.01; ***, *p* < 0.001. The uncropped bolts are shown in Supplementary Materials.

As a major methyltransferase responsible for m5C modification, we explored whether NSUN2 affects m5C modification levels in NPC. Overexpression of NSUN2 in CNE2 and 5-8F cells significantly increased m5C modification levels (Figure 4A), while NSUN2 knockdown markedly reduced these levels (Figure 4B).

To elucidate the mechanism by which NSUN2 promotes NPC via m5C modification, we initially collected 16 confirmed tumor suppressor genes and 27 potential tumor suppressor genes [40,41]. We identified 11 differentially expressed potential tumor suppressor genes in NPC by intersecting these genes with those from the differentially expressed genes in the NPC dataset GSE12452. Then, we utilized a gene set with altered m5C sites following NSUN2 silencing [42]. The intersection of the three datasets revealed that the tumor suppressor genes TP53 and PTEN were differentially expressed in NPC and may be regulated by NSUN2 through m5C modification (Figure 4C. Table S1). Western blot analysis revealed that NSUN2 overexpression had no significant impact on PTEN protein levels (Figure S2A), but suppressed TP53 protein levels in CNE2 and 5-8F cells (Figure 4D). Moreover, NSUN2 overexpression inhibited TP53 mRNA expression in CNE2 and 5-8F cells (Figure 4E), while NSUN2 silencing increased TP53 mRNA and protein levels (Figure 4F,G). Actinomycin D experiments demonstrated that NSUN2 overexpression decreased TP53 mRNA stability (Figure 4H). These results suggest that NSUN2 may regulate TP53 mRNA stability and expression through m5C modification, contributing to NPC progression.

### 3.4. NSUN2 Increases m5C Modification at the TP53 mRNA CDS 1228 Site, Reducing TP53 Expression

**Figure 5:**
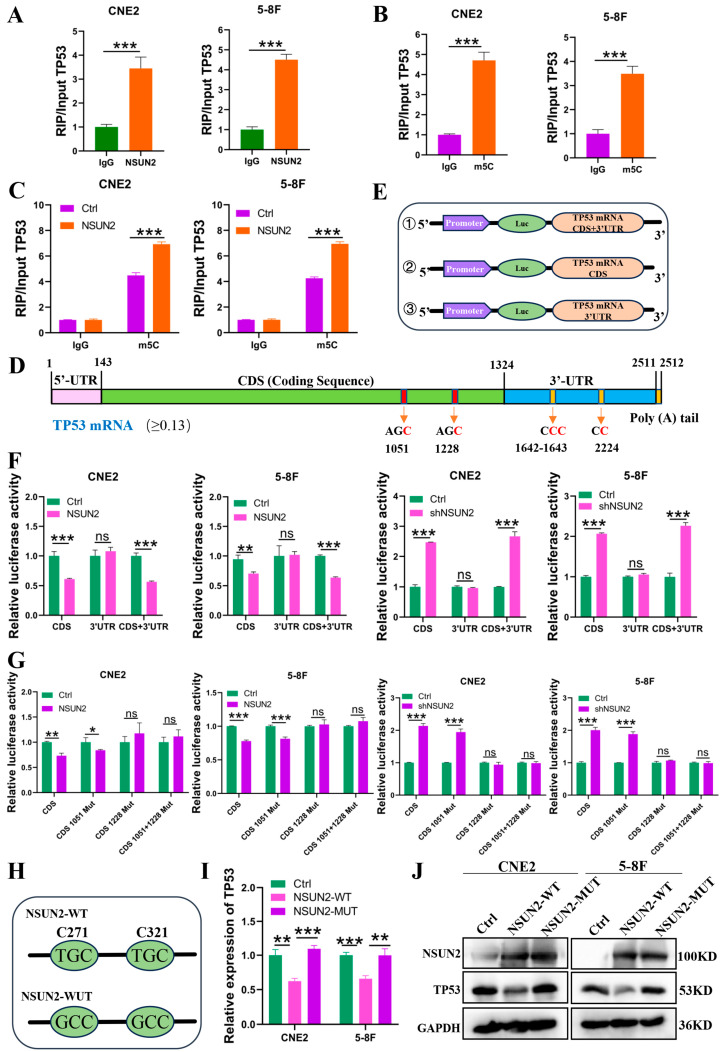
NSUN2 increases m5C modification at the CDS 1228 site of TP53 mRNA, reducing TP53 expression. (**A**) RIP assay confirming NSUN2 binding to TP53 mRNA. (**B**) RIP assay confirming m5C modification on TP53 mRNA. (**C**) RIP assay confirming NSUN2 can increase the m5C modification on TP53 mRNA. (**D**) Prediction of m5C modification sites on TP53 mRNA (shown in red font). (**E**) Construction of dual-luciferase reporter plasmids. (**F**) Dual-luciferase reporter assay confirming the NSUN2-regulated region on TP53 mRNA. (**G**) Dual-luciferase reporter assay confirming the NSUN2-regulated site on TP53 mRNA. (**H**) The two cysteine residues in NSUN2 were mutated to alanine. (**I**) Effect of NSUN2-MUT on TP53 mRNA expression. (**J**) Effect of NSUN2-MUT on TP53 protein expression. *, *p* < 0.05; **, *p* < 0.01; ***, *p* < 0.001. The uncropped bolts are shown in Supplementary Materials.

To further investigate how NSUN2 regulates TP53 via m5C modification, RIP experiments showed that NSUN2 binds to TP53 mRNA (Figure 5A). M5C-RIP experiments further demonstrated that m5C modification exists on TP53 mRNA. Notably, NSUN2 overexpression increased m5C modification on TP53 mRNA, whereas NSUN2 knockdown reduced it, revealing that NSUN2 decreases TP53 mRNA stability through m5C modification, thus promoting NPC development (Figure 5B,C and Figure S2B).

To investigate the specific regions and sites through which NSUN2 regulates TP53, we utilized iRNA-m5C and RNA m5C Finder to predict m5C modification sites on TP53 mRNA. Multiple m5C sites were identified, predominantly located in the CDS region and the 3′ UTR of TP53 mRNA, with the highest m5C modification score found in the CDS region (Figure 5D). To further validate the specific regions where NSUN2 regulates TP53 mRNA, we constructed three dual-luciferase reporter plasmids: PGL3-Promoter/TP53 mRNA CDS + 3′ UTR, PGL3-Promoter/TP53 mRNA CDS, and PGL3-Promoter/TP53 mRNA 3′ UTR (Figure 5E). These plasmids were co-transfected with a control vector into NPC cells, and dual-luciferase assays were performed. The results demonstrated that NSUN2 predominantly regulates the TP53 mRNA CDS region, consistent with the predicted modification sites (Figure 5F). Additionally, we predicted that the m5C sites on TP53 mRNA correspond to cytosine positions within AGC motifs. To investigate NSUN2’s regulation of these m5C sites, we employed mutagenesis to construct plasmids with single mutations at CDS positions 1051 and 1228, as well as double mutations (Figure S2C). Our findings showed that NSUN2 primarily modifies TP53 mRNA at the CDS 1228 site (Figure 5G). Furthermore, we introduced mutations at two sites critical for NSUN2 enzymatic activity, including C271 (mediating methyl group release) and C321 (catalytic site) (Figure 5H) [43]. NSUN2-MUT showed no significant effect on TP53 mRNA or protein expression levels (Figure 5I,J).

### 3.5. TP53 Knockdown Reverses the Suppressive Effects of NSUN2 Knockdown on NPC Cells

**Figure 6:**
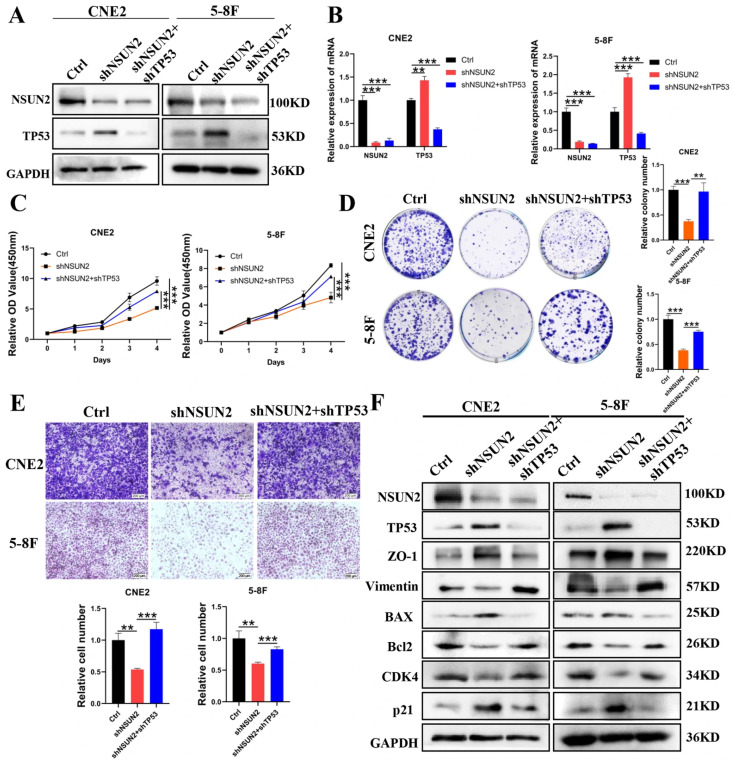
TP53 knockdown reverses the phenotypic effects of NSUN2 knockdown in nasopharyngeal carcinoma cells. (**A**) Western blot analysis of TP53 protein levels in CNE2 and 5-8F cells after decreasing TP53 expression. (**B**) qPCR analysis of TP53 mRNA levels in CNE2 and 5-8F cells after reducing TP53 expression. (**C**) CCK-8 assay evaluating the effect of decreasing TP53 expression on cell proliferation in CNE2 and 5-8F cells. (**D**) Clonogenic assay assessing the effect of decreasing TP53 expression on colony formation in CNE2 and 5-8F cells. (**E**) Transwell assay evaluating the effect of reducing TP53 expression on cell invasion. (**F)** Western blot analysis of downstream molecular effects of TP53 knockdown. **, *p* < 0.01; ***, *p* < 0.001. The uncropped bolts are shown in Supplementary Materials.

The results presented above confirm that NSUN2 promotes the malignant biological phenotype of NPC cells, functioning as an oncogene. Mechanistically, NSUN2 negatively regulates TP53 expression through m5C modification. To further explore the role of TP53 in NSUN2-mediated biological functions, we conducted reversal experiments. In CNE2 and 5-8F cells with NSUN2 knockdown, TP53 expression was upregulated. Knockdown of TP53 in NSUN2-knockdown cells reversed the elevated TP53 levels induced by NSUN2 knockdown (Figure 6A,B). CCK-8 assays revealed that NSUN2 knockdown reduced cell proliferation, while TP53 knockdown reversed this effect (Figure 6C). Colony formation assays similarly showed that NSUN2 knockdown led to a decrease in colony number, which was subsequently restored upon TP53 knockdown (Figure 6D). Additionally, flow cytometry analysis demonstrated that NSUN2 knockdown increased apoptosis, and this effect was reversed by decreasing TP53 expression (Figure S3A). Wound healing and invasion assays showed that decreasing TP53 expression enhanced the migration and invasion capabilities of CNE2 and 5-8F cells (Figure S3B and Figure 6E). Western blot analysis further indicated that NSUN2 knockdown affected the expression of EMT-related genes, such as ZO-1 and vimentin, apoptosis-related genes, such as BAX and Bcl2, and cell cycle-related genes, including CDK4 and p21. Reduction of TP53 expression counteracted these effects (Figure 6F). Collectively, these findings suggest that TP53 knockdown can partially reverse the suppressive effects of NSUN2 knockdown on the malignant phenotype of NPC cells.

### 3.6. NSUN2 Knockdown Inhibits NPC Growth by Regulating In Vivo TP53 Expression

**Figure 7:**
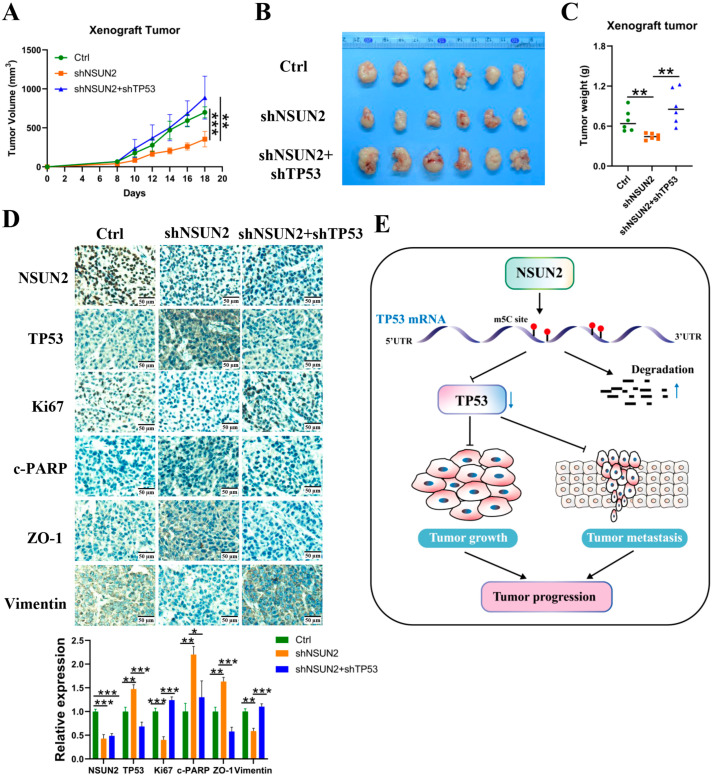
In vivo validation of the effect of NSUN2 on nasopharyngeal carcinoma cell proliferation. (**A**) Growth curve of nude mice. (**B**) Tumor volumes in different groups of nude mice. (**C**) Tumor weights in different groups of nude mice. (**D**) Detection of tumor tissue markers (Scale bar: 50 µm). (**E**) Schematic diagram illustrating that NSUN2 negatively regulates TP53 to promote the malignant progression of nasopharyngeal carcinoma. *, *p* < 0.05; **, *p* < 0.01; ***, *p* < 0.001.

To further investigate the role of NSUN2 in tumor growth in NPC in vivo and to assess the contribution of TP53 to NSUN2-driven tumorigenesis, we injected stable CNE2 cell lines expressing shPLVX (control), shNSUN2, and shNSUN2 + shTP53 into the subcutaneous tissue of nude mice. We measured tumor size at defined time points. Compared to the control group, the knockdown of NSUN2 significantly inhibited NPC growth. Additionally, the simultaneous knockdown of TP53 reversed the inhibitory effect of NSUN2 knockdown on tumor growth, indicating that NSUN2 promotes tumor growth by suppressing TP53 expression (Figure 7A–C and Figure S4A,B).

Immunohistochemical analysis of the tumor tissues of nude mice revealed that the knockdown of NSUN2 led to increased TP53 expression, reduced Ki67 and vimentin expression (a proliferation markers), and increased c-PARP (an apoptosis marker) and ZO-1 expression. Conversely, decreasing TP53 expression in NSUN2-knockdown tumors reversed the reduction in Ki67 and vimentin expression and the elevation in c-PARP and ZO-1 expression (Figure 7D).

## 4. Discussion

In this study, we investigated the expression of the m5C methyltransferase NSUN2 in NPC. Our findings indicate that NSUN2 is highly expressed in NPC and is involved in promoting cell proliferation and clonogenicity, while inhibiting apoptosis. Additionally, NSUN2 enhances migration, invasion, and tumor growth in vivo. These results suggest that NSUN2 may act as a potential oncogene in NPC. Furthermore, numerous studies support role of NSUN2 as a tumor marker, as it is overexpressed in various cancers, including prostate, liver, stomach cancers, and esophageal squamous cell carcinoma [17,21,23,27], contributing to the promotion of malignant biological phenotypes such as metabolism, proliferation, invasion, immune response, cell death, and differentiation [16,44]. For example, in hepatocellular carcinoma (HCC), NSUN2 promotes cell proliferation, migration, and tumor growth, while in cervical cancer, NSUN2 increases the m5C modification of LRRC8A, thereby inhibiting apoptosis and promoting cisplatin resistance [22]. Moreover, high NSUN2 expression has been linked to resistance to gefitinib and tumor recurrence in non-small-cell lung cancer [45].

Our study demonstrates that NSUN2 binds to TP53 mRNA and primarily regulates the CDS region of TP53 mRNA, specifically modifying the 1228 site. This modification decreases TP53 mRNA stability, inhibits TP53 mRNA and protein expression, and promotes NPC progression. As a major m5C methyltransferase, NSUN2 regulates downstream target genes through m5C modifications, contributing to tumorigenesis. For example, in endometrial cancer, NSUN2 increases m5C modifications on SLC7A11 mRNA, enhancing its stability and expression, reducing lipid peroxidation, and inhibiting ferroptosis [26]. Similarly, NSUN2 methylates LINC00324 via m5C modifications, increasing its stability and expression, which promotes glioma angiogenesis [46]. NSUN2 also enhances m5C modifications on three prime repair exonuclease 2 mRNA, promoting its expression, inhibiting cGAS/STING activation, and facilitating tumor development [25]. Additionally, in the case of tumor suppressor genes, NSUN2 also decrease the mRNA stability of p57^Kip2^ through m5C modification, thereby facilitating the progression of gastric cancer [19]. These findings highlight NSUN2′s critical role in tumorigenesis by modulating downstream target genes through m5C modifications.

TP53 is involved in various biological processes such as apoptosis, metabolism, and DNA damage repair, thereby inhibiting tumorigenesis [30]. In our study, we found that TP53 reversed the malignant phenotype mediated by NSUN2 in CNE2 and 5-8F cells, demonstrating that TP53 plays a crucial role in NSUN2-mediated malignant progression in NPC. Increasing evidence suggests that TP53 is essential in tumor suppression and inhibits tumor progression. For example, the activation of TP53 expression by oridonin inhibited TCF4 transactivation, thereby increasing the ROS levels in tumor cells, promoting the release of Ca^2+^, and inhibiting the occurrence of colorectal cancer [47]. In HCC, leucine rich repeat containing G protein-coupled receptor 5 bound and blocked the nuclear translocation of programmed cell death protein 5, reducing the stability of TP53 and promoting the degradation of TP53, thus promoting EMT and the drug resistance of HCC cells to doxorubicin [48]. This research shows that the tumor suppressor gene TP53 is critical in tumors. However, further validation with larger clinical samples is needed to examine the correlation between TP53 mRNA m5C modification levels, NSUN2 expression, and patient prognosis.

Given the established association between NSUN2 overexpression and poor prognosis in NPC, coupled with the ongoing challenge of TP53 undruggability, our study reveals that NSUN2 directly represses TP53 expression via m5C-dependent mRNA methylation, thereby accelerating the progression of NPC. This mechanistic insight opens two therapeutic avenues: By targeting the catalytic domain of NSUN2 responsible for site-specific m5C deposition on TP53 mRNA, we can develop selective inhibitors to block this oncogenic modification and hinder tumor progression; alternatively, designing small-molecule or peptide-based NSUN2 degraders offers a complementary strategy to suppress NSUN2-driven malignancy, collectively expanding the therapeutic landscape for NPC.

## 5. Conclusions

Our study demonstrates that NSUN2 is upregulated in NPC and associated with poor prognosis. Functionally, NSUN2 promotes malignant phenotypes including proliferation, migration, and invasion while suppressing apoptosis. Mechanistically, NSUN2 mediates m5C modification at the CDS 1228 site of TP53 mRNA, leading to its destabilization and downregulation. Additionally, TP53 knockdown reverses the tumor-suppressive effects of NSUN2 knockdown in vitro and in vivo. These results establish the NSUN2/TP53 axis as a potential therapeutic target in NPC (Figure 7E).

## Figures and Tables

**Table 1:** Sequences used in this study.

Targets	Sequence
siRNA	
siNSUN2#1	5′-GGAGAACAAGCTGTTCGAG-3′
siNSUN2#2	5′-GAGATCCTCTTCTATGATC-3′
shRNA	
shNSUN2	F: GATCCGAAGCATCGTGCTGAAGTACTCGAGTACTTCAGCACGATGCTTCTTTTTG R: AATTCAAAAAGAAGCATCGTGCTGAAGTACTCGAGTACTTCAGCACGATGCTTCG
shTP53	F: GATCCGACTCCAGTGGTAATCTACCTCGAGGTAGATTACCACTGGAGTCTTTTTG R: AATTCAAAAAGACTCCAGTGGTAATCTACCTCGAGGTAGATTACCACTGGAGTCG
TP53 related primer	
TP53-CDS	F: CGCCGTGTAATTCTAGGAAACTACTTCCTGAAAACAA R: CCGCCCCGACTCTAGTTCTGTCTTGAACATGAGTTT
TP53-3′UTR	F: CGCCGTGTAATTCTAGAGGACTTCCATTTGCTTT R: CCGCCCCGACTCTAGATATAAAAATGGGATATAAAAAGGG
TP53-CDS + 3′UTR	F: GCCGTGTAATTCTAGAAGAGAATCTCCGCAAGAAA R: CCGCCCCGACTCTAGATATAAAAATGGGATATAAAAAGGG
TP53 CDS 1051 Mut	F: GCTGCCCCCAGGGAGTACTAAGCGAGCACTGCCCAA R: CAGTGCTCGCTTAGTACTCCCTGGGGGCAGCTCGTG
TP53 CDS 1228 Mut	F: GGAGCCAGGGGGGAGTAGGGCTCACTCCAGCCACCT R: GCTGGAGTGAGCCCTACTCCCCCCTGGCTCCTTCCC
TP53 CDS 1051 + 1228 Mut	F: GCTGCCCCCAGGGAGTACTAAGCGAGCACTGCCCAA R: GCTGGAGTGAGCCCTACTCCCCCCTGGCTCCTTCCC
Primer	
NSUN2	F: 5′-AAGAAAGATGGCGTGTGTGG-3′ R: 5′-TATTCAGCAGCACATTCCGC-3′
TP53	F: 5′-CTCAGATAGCGATGGTCTGG-3′ R: 5′-CTGTCATCCAAATACTCCACAC-3′
GAPDH	F: 5′-CAACGGATTTGGTCGTATTGG-3′ R: 5′-TGACGGTGCCATGGAATTT-3′
18S RNA	F: 5′-TCTTAGCTGAGTGTCCCGCG-3′ R: 5′-ATCATGGCCTCAGTTCCGAA-3′

**Table 2:** Clinical correlation between NSUN2 expression and nasopharyngeal carcinoma tissue samples.

Variable Features	NSUN2 Expression		*p*
	Low Expression (16)	High Expression (60)	
Age (year)			
≤53	7 (43.75%)	32 (53.33%)	0.4956
>53	9 (56.25%)	28 (46.67%)	
Gender			
Male (*n* = 58)	11 (68.75%)	47 (78.33%)	0.4231
Female (*n* = 18)	5 (31.25%)	13 (21.67%)	
Clinical stages (*n* [%])			
Ⅰ–Ⅱ	12 (75%)	7 (11.67%)	*** *p* < 0.0001
Ⅲ–Ⅳ	4 (25%)	53 (88.33%)	
Tumor size (*n* [%])			
T1–2	13 (81.25%)	18 (30%)	*** *p* < 0.001
T3–4	3 (18.75%)	42 (70%)	
Lymph node metastasis (*n* [%])			
no	8 (50%)	3 (5%)	*** *p* < 0.0001
yes	8 (50%)	57 (95%)	

## Data Availability

Data will be made available on request. For requesting data, please write to the corresponding author.

## Supplementary Materials

The following supporting information can be downloaded at: https://www.mdpi.com/article/10.3390/cancers17243950/s1, Figure S1: Molecular analysis of m5C modification in NPC; Figure S2: Effect of NSUN2 on PTEN expression and m5C modification on TP53 and identification of three dual-luciferase reporter vectors; Figure S3: Effect of decreasing TP53 expression on NSUN2 knockdown-mediated inhibitory roles; Figure S4: Effect of decreasing TP53 expression on NSUN2 knockdown-mediated inhibitory roles on proliferation in vivo; Table S1: Screening list of potential downstream target genes of NSUN2.
