# Supplementary material for: NSUN2 Negatively Regulates TP53 mRNA Stability to Promote the Malignant Progression of Nasopharyngeal Carcinoma
Source: Cancers (Basel). 2025 Dec 10;17(24):3950. doi: 10.3390/cancers17243950 (PMC12731150; doi:10.3390/cancers17243950)
Supplement: Supplementary file 1 [file cancers-17-03950-s001.zip › Original image for checking.pdf]

Original blots in this manuscript

1.Original blots used in Figure 1C

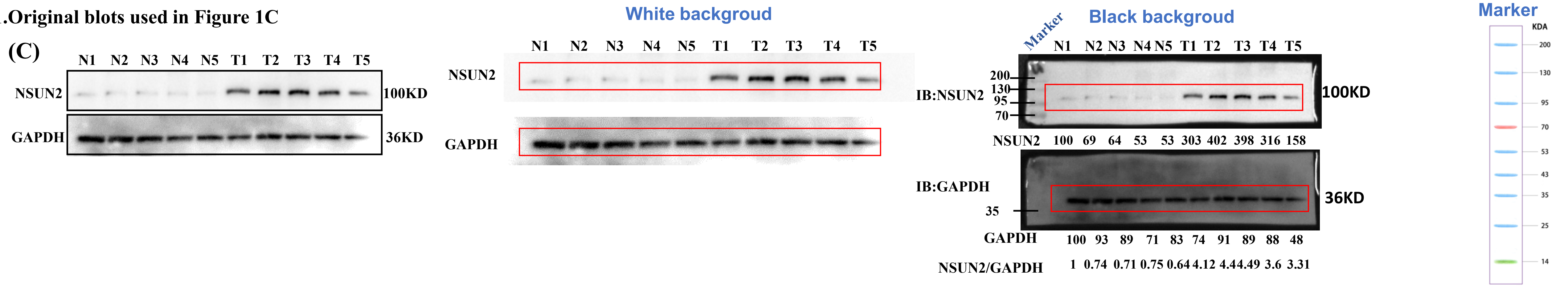

2.Original blots used in Figure 2A

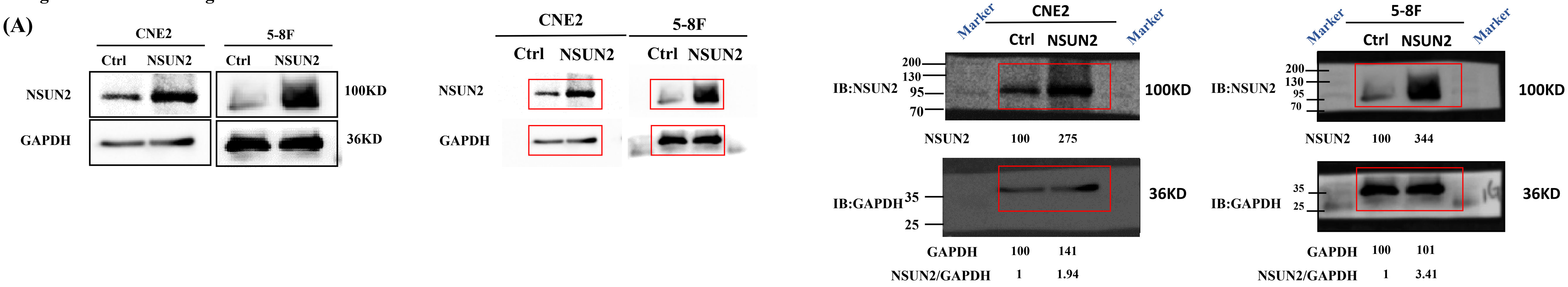

3.Original blots used in Figure 3A

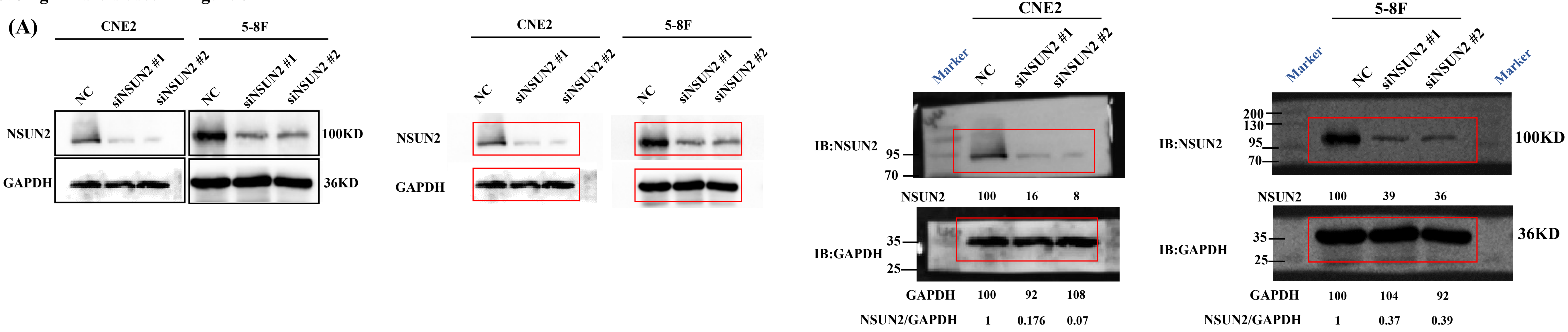

4.Original blots used in Figure 4A and 4B

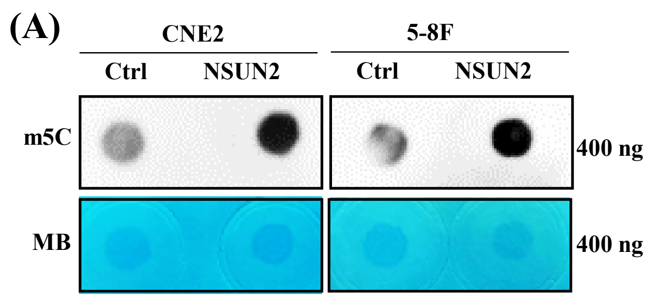

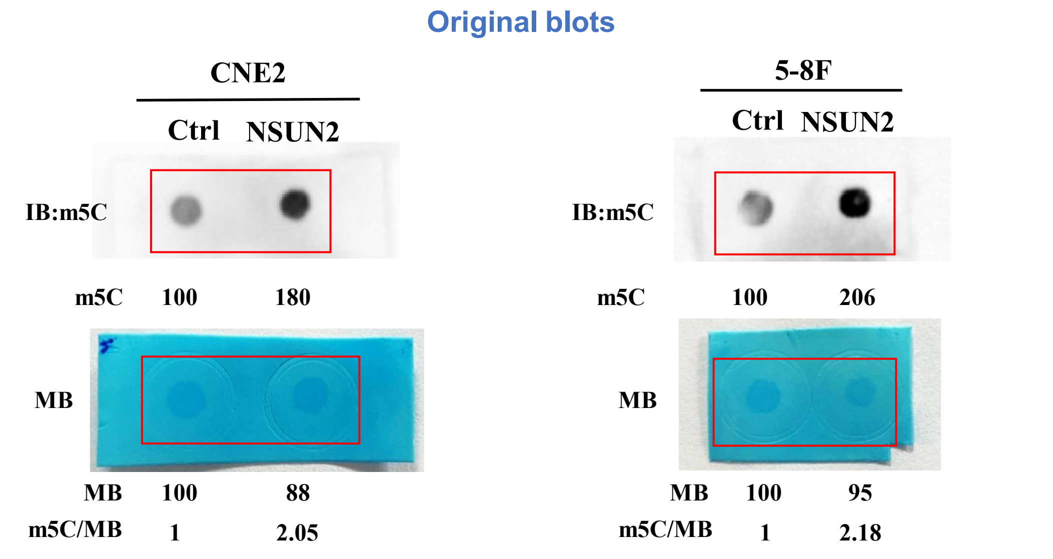

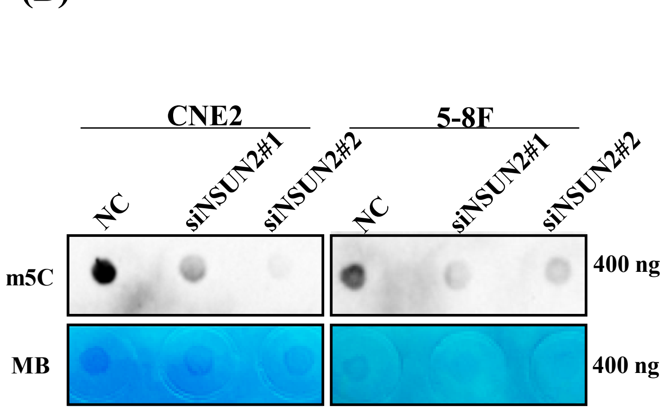

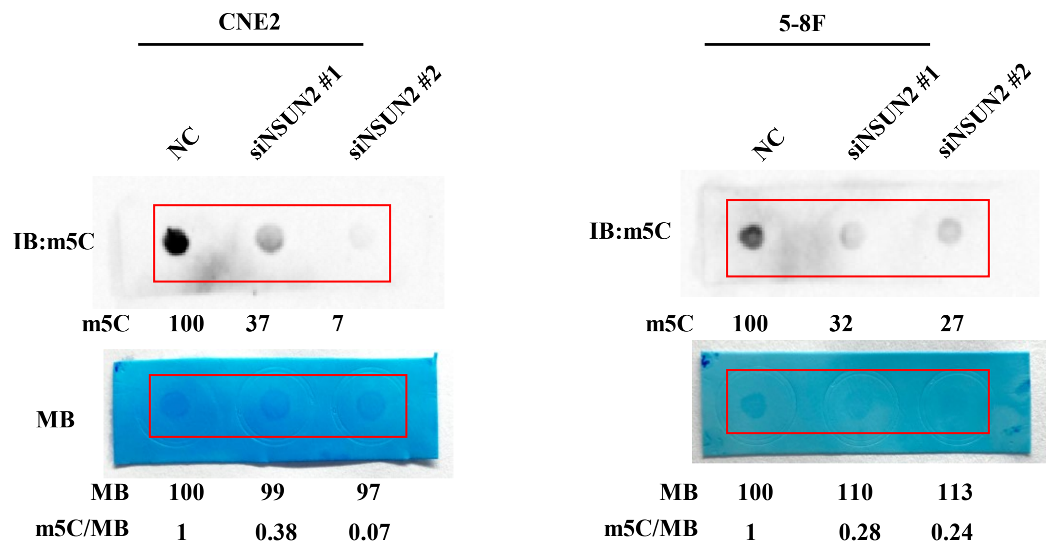

Original blots used in Figure 4D and 4F

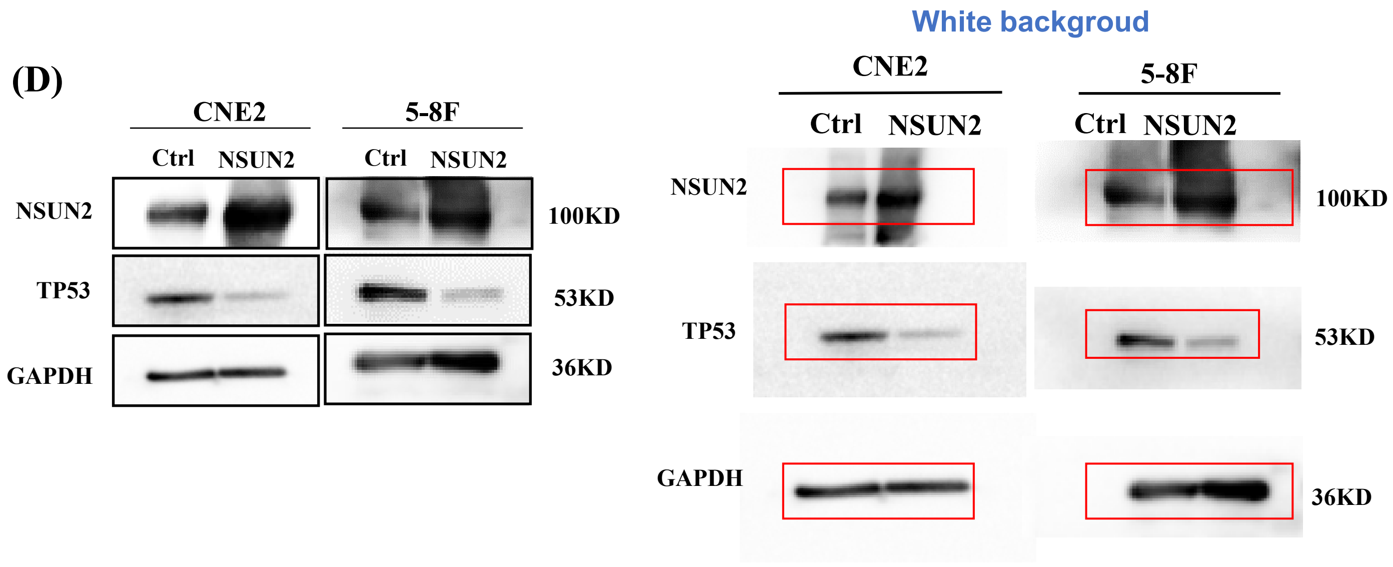

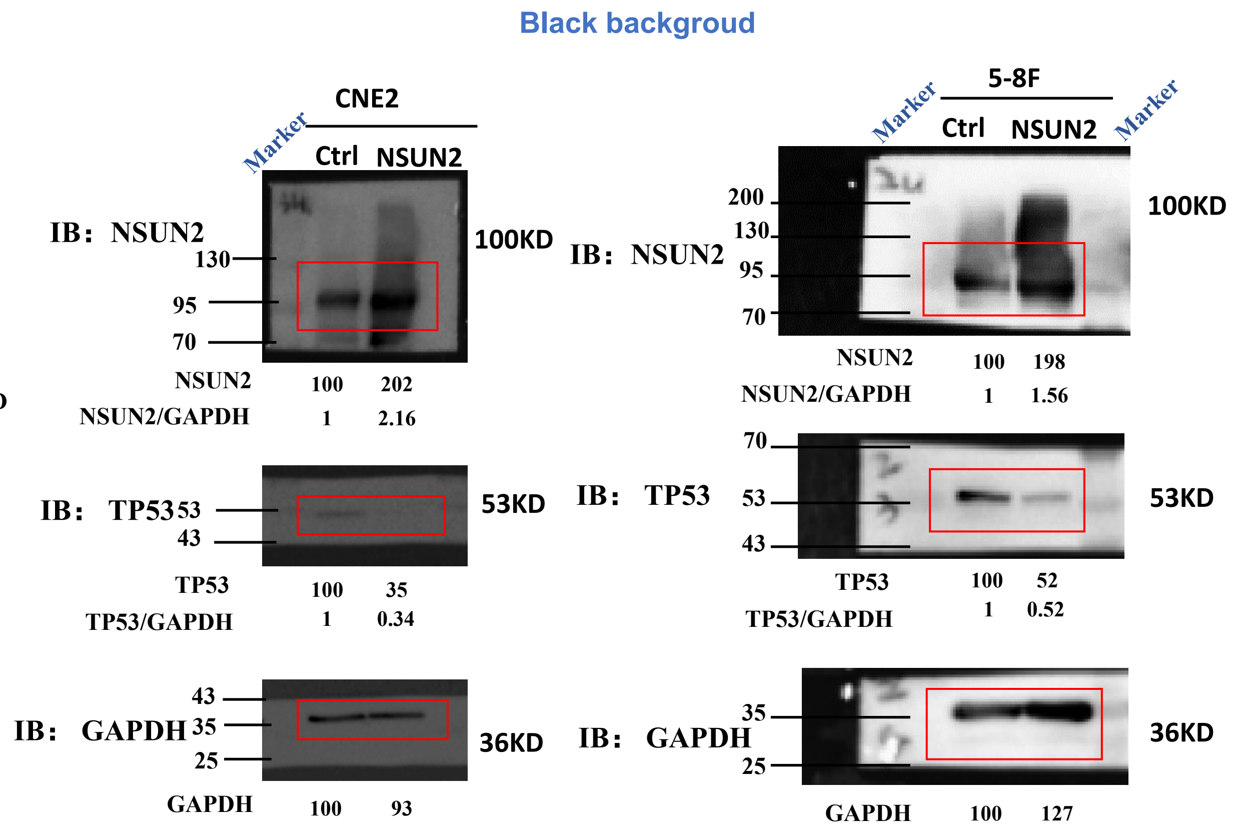

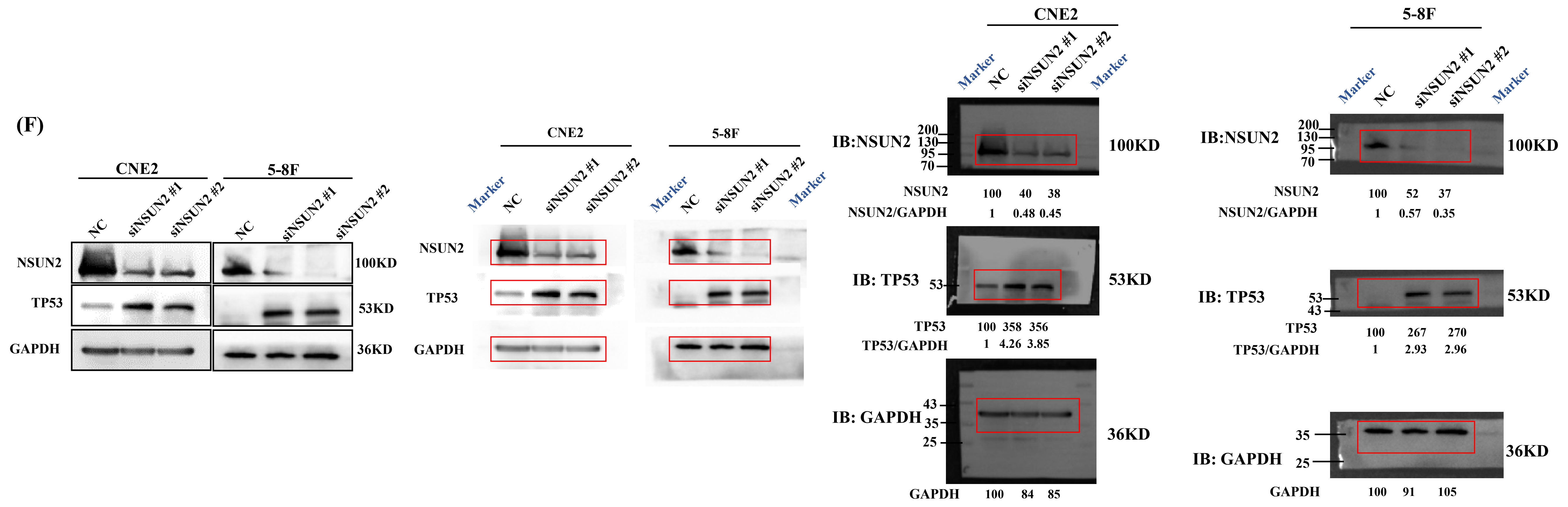

5.Original blots used in Figure 6A and Figure 6F

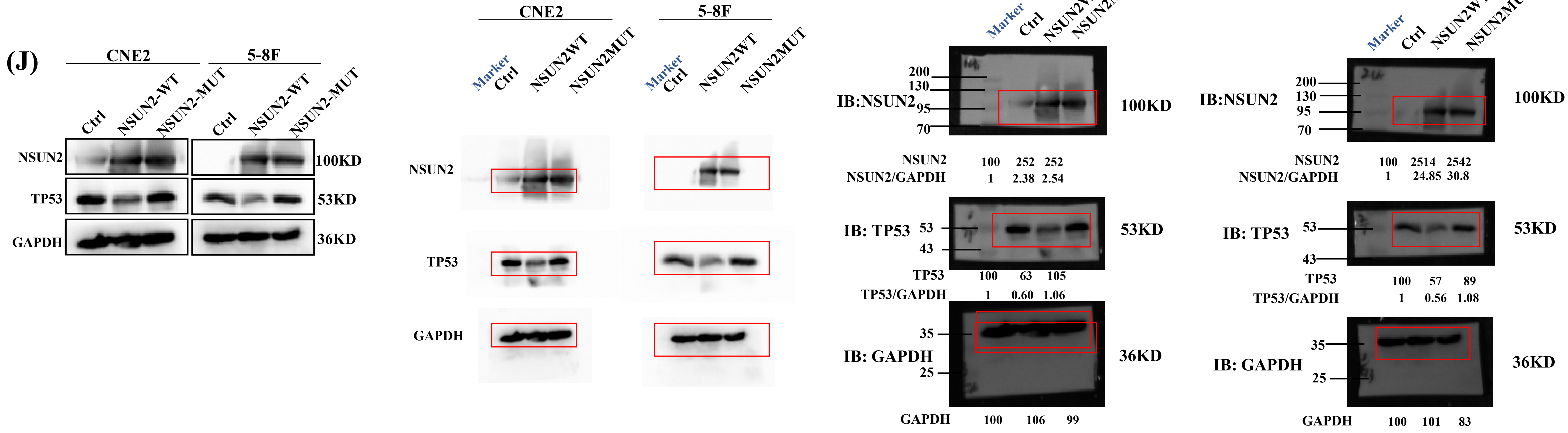

5.Original blots used in Figure 6A and Figure 6F

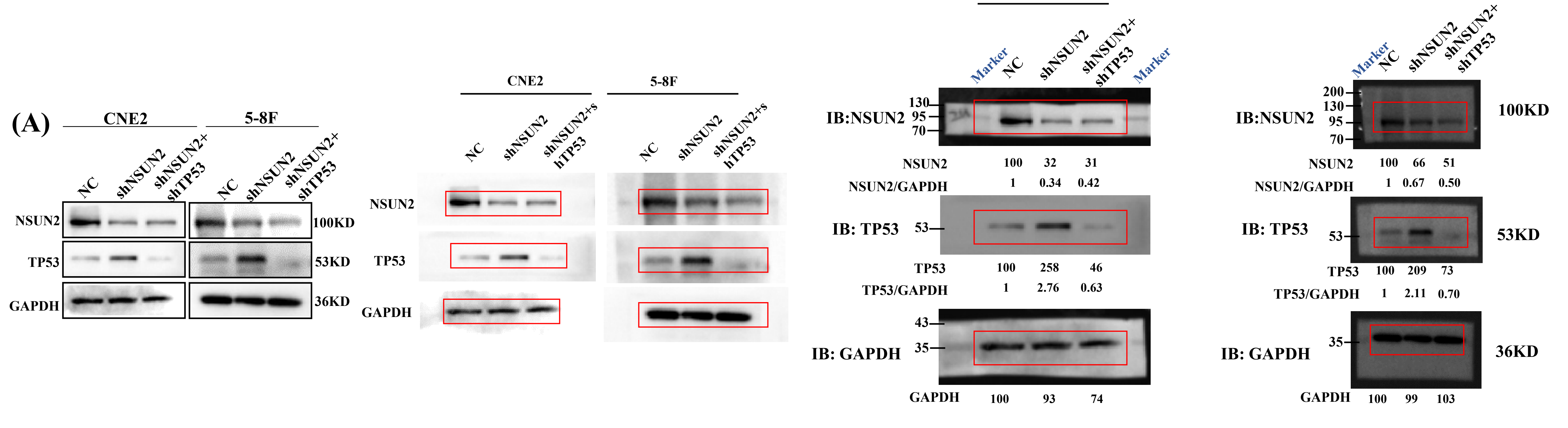

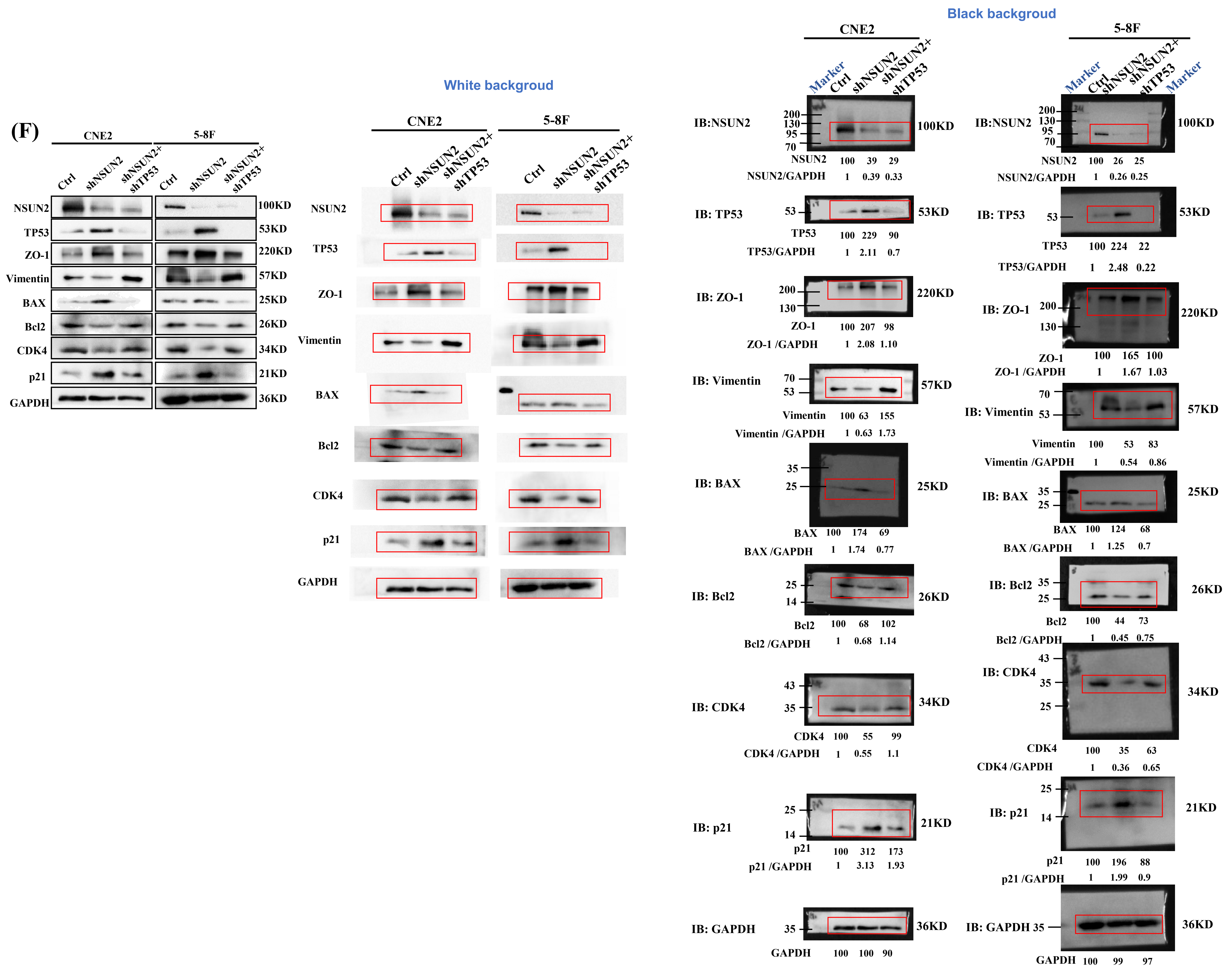

(F)
CNE2
5-8F
Ctrl
shNSUN2
shNSUN2+shTP53
NSUN2
TP53
ZO-1
Vimentin
BAX
Bcl2
CDK4
p21
GAPDH
100KD
53KD
220KD
57KD
25KD
26KD
34KD
21KD
36KD
White backgroud
Black backgroud
Marker
IB:NSUN2
200
130
95
70
NSUN2 100 39 29
NSUN2/GAPDH 1 0.39 0.33
NSUN2 100 26 25
NSUN2/GAPDH 1 0.26 0.25
IB: TP53
53
TP53 100 229 90
TP53/GAPDH 1 2.11 0.7
TP53 100 224 22
TP53/GAPDH 1 2.48 0.22
IB: ZO-1
ZO-1 100 207 98
ZO-1 /GAPDH 1 2.08 1.10
ZO-1 100 165 100
ZO-1 /GAPDH 1 1.67 1.03
IB: Vimentin
Vimentin 100 63 155
Vimentin /GAPDH 1 0.63 1.73
Vimentin 100 53 83
Vimentin /GAPDH 1 0.54 0.86
IB: BAX
35
25
BAX 100 174 69
BAX /GAPDH 1 1.74 0.77
BAX 100 124 68
BAX /GAPDH 1 1.25 0.7
IB: Bcl2
14
Bcl2 100 68 102
Bcl2 /GAPDH 1 0.68 1.14
Bcl2 100 44 73
Bcl2 /GAPDH 1 0.45 0.75
IB: CDK4
43
CDK4 100 55 99
CDK4 /GAPDH 1 0.55 1.1
CDK4 100 35 63
CDK4 /GAPDH 1 0.36 0.65
IB: p21
p21 100 312 173
p21 /GAPDH 1 3.13 1.93
p21 100 196 88
p21 /GAPDH 1 1.99 0.9
IB: GAPDH
GAPDH 100 100 90
GAPDH 100 99 97
