# Supplementary material for: NSUN2 Negatively Regulates TP53 mRNA Stability to Promote the Malignant Progression of Nasopharyngeal Carcinoma
Source: Cancers (Basel). 2025 Dec 10;17(24):3950. doi: 10.3390/cancers17243950 (PMC12731150; doi:10.3390/cancers17243950)
Supplement: Supplementary file 1 [file cancers-17-03950-s001.zip › Supplementary Figures.pdf]

Supplementary Figures

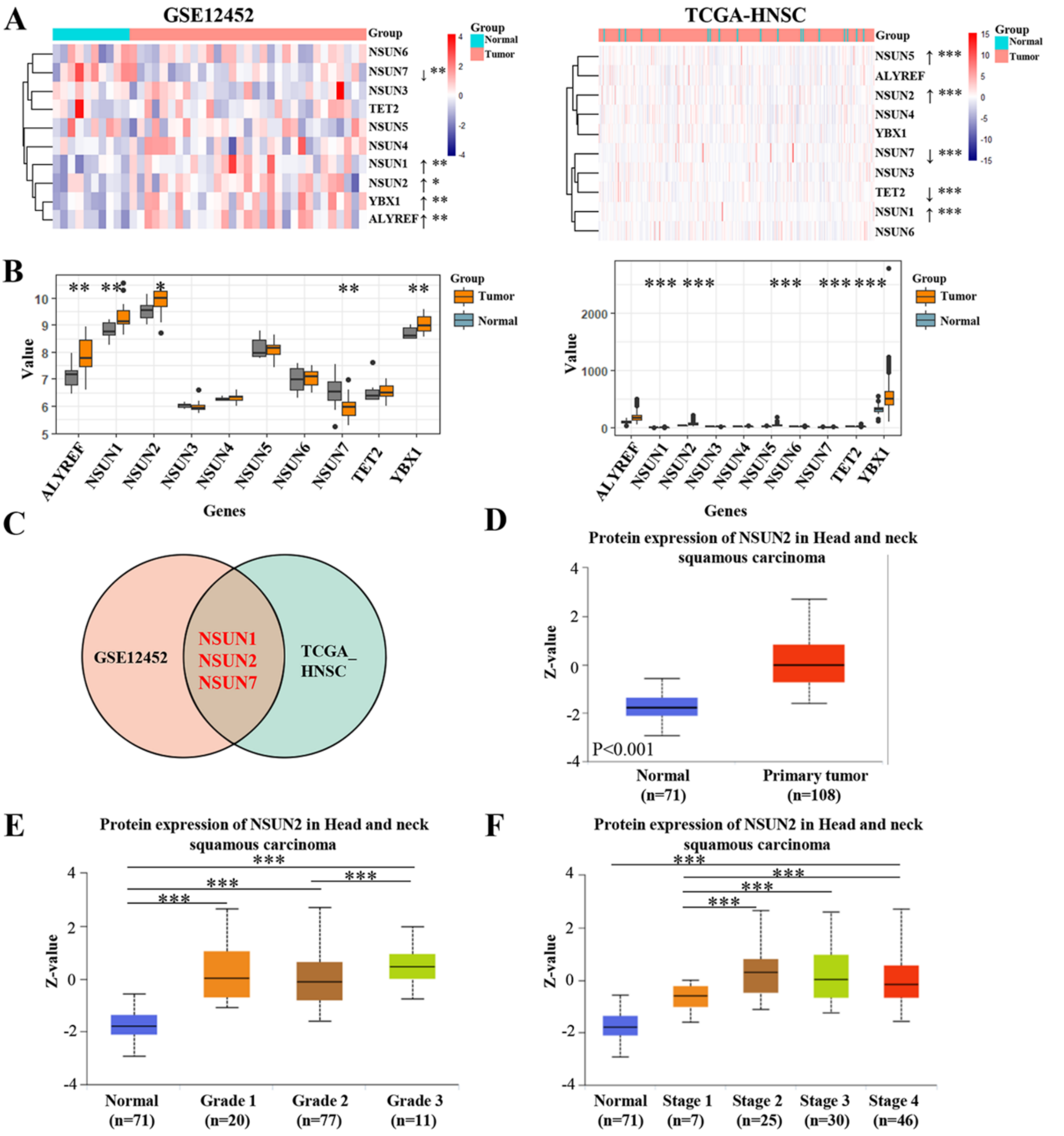

Figure S1. Molecular analysis of m5C modification in NPC. (A) Heatmap of m5C modification-related gene expression in the GEO dataset GSE12452 and TCGA-HNSC dataset. (B) Box plot showing the expression of m5C modification-related genes in the GEO dataset GSE12452 and TCGA-HNSC dataset. (C) Venn diagram of overlapping m5C modification-related genes between the two datasets. (D) Protein expression level of NSUN2 in TCGA dataset. (E) Correlation between NSUN2 expression and NPC progression in TCGA-HNSC. (F) Correlation between NSUN2 expression and NPC staging in TCGA-HNSC. *, $P < 0.05$; **, $P < 0.01$; ***, $P < 0.001$.

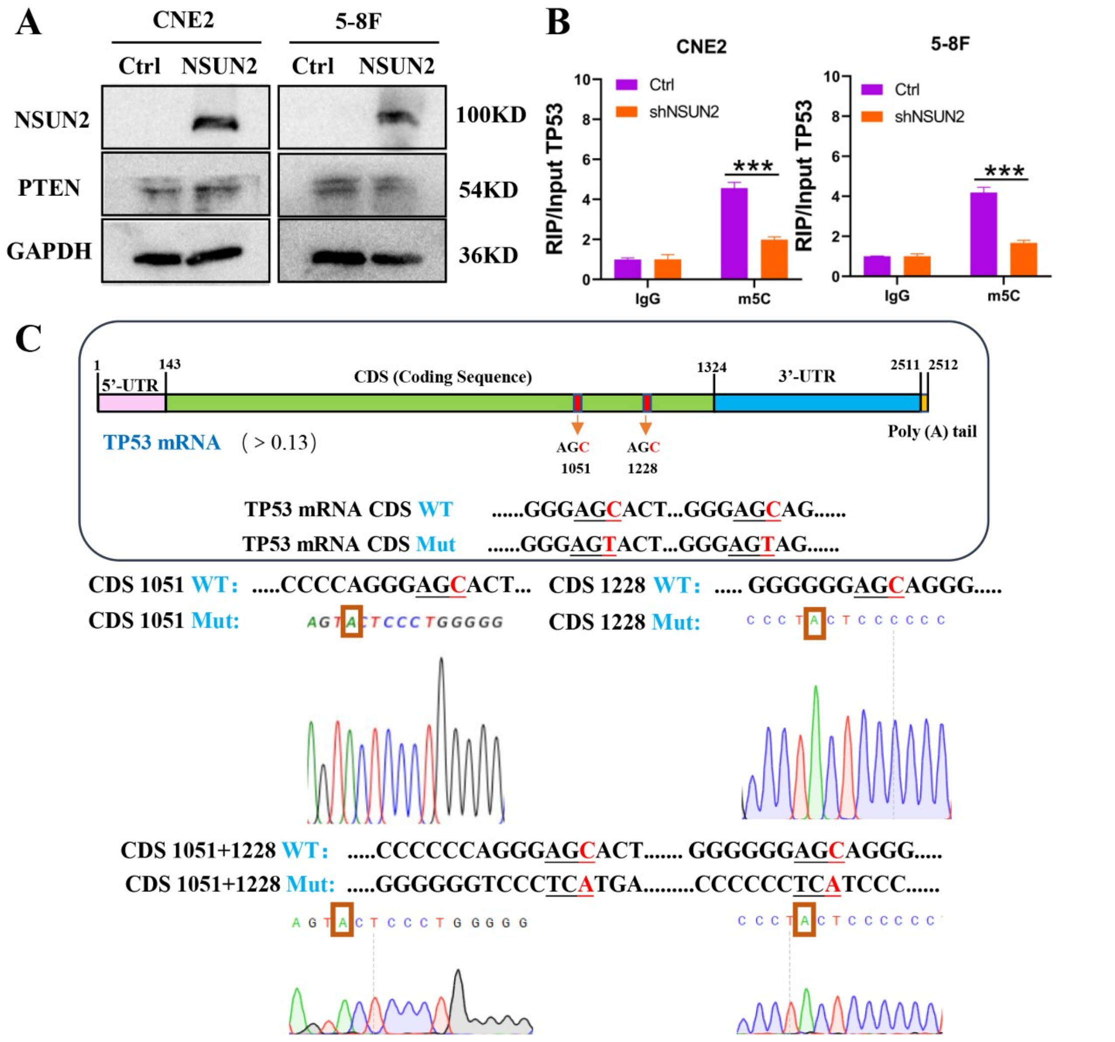

Figure S2. Effect of NSUN2 on PTEN expression and m5C modification on TP53 and identification of three dual-luciferase reporter vectors.

(A) Effect of NSUN2 overexpression on PTEN protein levels. (B) RIP assay confirming shNSUN2 can decrease the m5C modification on TP53 mRNA. (C) Identification of three dual-luciferase reporter vectors fused with mutated TP53 CDS by sequencing.

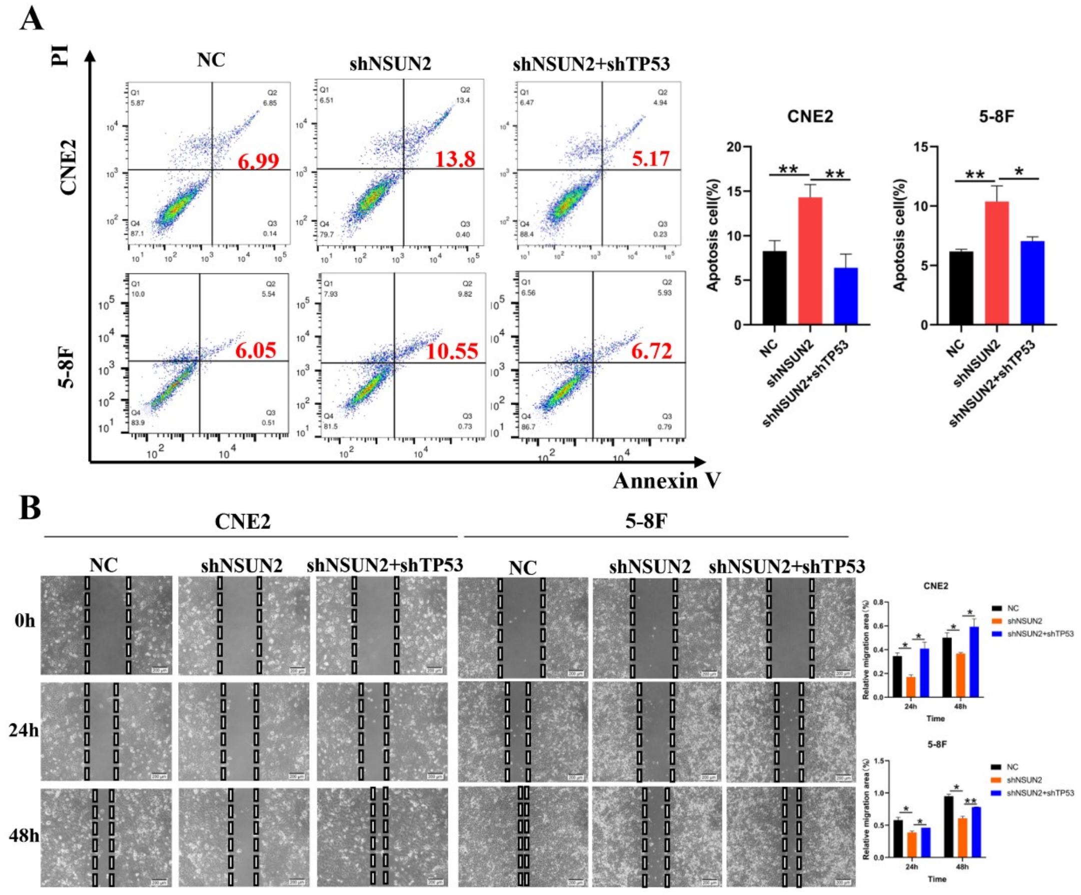

Figure S3. Effect of decreasing TP53 expression on NSUN2 knockdown-mediated inhibitory roles. (A) Apoptosis assay evaluating the effect of decreasing TP53 expression on cell apoptosis. (B) Scratch assay assessing the effect of decreasing TP53 expression on cell migration.

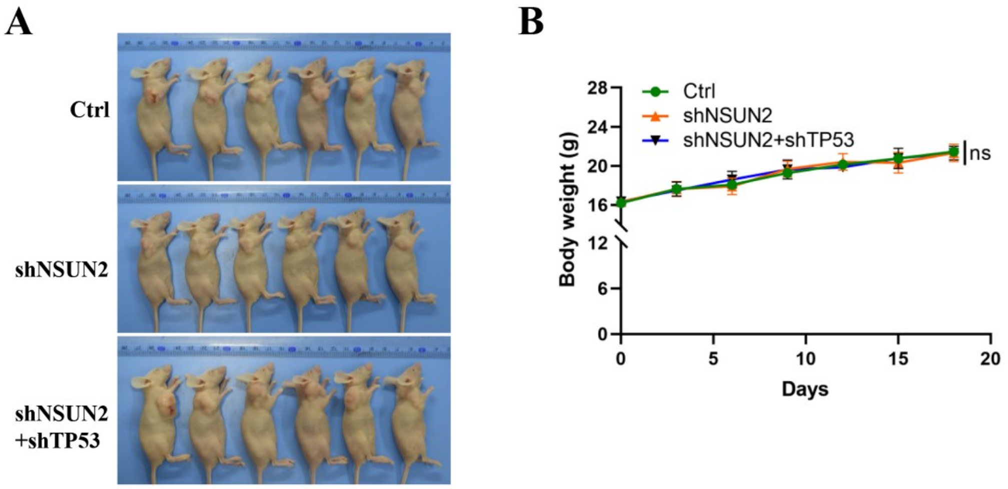

Figure S4. Effect of decreasing TP53 expression on NSUN2 knockdown-mediated inhibitory roles on proliferation *in vivo*. (A) Images of mice. (B) Body weight of mice.
